# The association of sleep duration and metabolic syndrome in the Bandare-Kong cohort study, a cross-sectional survey (finding from PERSIAN cohort study)

**DOI:** 10.1186/s13098-021-00737-1

**Published:** 2021-10-20

**Authors:** Shideh Rafati, Maryam Isheh, Abnoos Azarbad, Farhad Ghadiri Soufi, Arash Rahimi, Masoumeh Kheirandish

**Affiliations:** 1 Social Determinants in Health Promotion Research Center, Hormozgan University of Medical Sciences, Bandar Abbas, Iran; 2 Student Research Committee, Faculty of Medicine, Hormozgan University of Medical Sciences, Bandar Abbas, Iran; 3 Department of Pharmacology, Faculty of Pharmacy, Eastern Mediterranean University, via Mersin 10, Famagusta, North Cyprus Turkey; 4 Endocrinology and Metabolism Research Center, Hormozgan University of Medical Sciences, Bandar Abbas, Iran

**Keywords:** Metabolic syndrome, Sleep disorder, Generalized analyzed model, Prospective Epidemiological Research Studies in IrAN (PERSIAN)

## Abstract

**Background:**

A variety of health problems, such as metabolic syndrome (MetS), have been linked to sleep disorders. While numerous epidemiological studies have shown a U-shaped relationship between sleep duration and poor health outcomes, the results were limited and inconsistent. This study was designed to evaluate the relationship between sleep duration and MetS.

**Methods:**

This population-based study was conducted on the participants aged 35–70 of Bandare-Kong Non-Communicable Diseases (BKNCD) Cohort Study, a part of Prospective Epidemiological Research Studies in IrAN (PERSIAN). MetS was diagnosed according to the National Cholesterol Education Program (NCEP) criteria and the Iranian-specific cut-off for waist circumference (≥ 95 cm). Sleep information was extracted through a standard questionnaire based on self-reported information. Data were analyzed by R software using generalized additive models (GAMs). A statistically significant level was considered as P < 0.05.

**Results:**

A total of 3695 participants were included in the analyses. The mean age was 48.05 years (SD 9.36), and 2067 (55.9%) were female. The estimated Prevalence of MetS was 35.9%, and women appeared to be more likely to have MetS than men (P < 0.001). There was a non-linear and linear association between sleep duration and the risk of MetS in women and men, respectively. The lowest risk was observed among those with 7–7.5 h of sleep duration per night.

**Conclusion:**

Long sleep duration was associated with increased risk of MetS and higher MetS severity score in both genders, while the short sleep duration increased the risk of Mets as well as MetS severity score just in women. The longitudinal studies would be suggested to assess the relationship between sleep quality and quantity components and MetS.

## Introduction

Sleep is an essential lifestyle element that can be surveyed as a significant preventive measure, an indicator to evaluate an individual’s current health condition or a health outcome that may lead to other health complications [1]. Therefore, to develop healthy sleep quality, enhance daytime alertness and overall well-being, sleep-specific guidelines have advised adults to sleep 7 to 8 h per night. Guidelines also recommend adults to follow a healthy sleep regimen, such as narrowing their daytime naptimes to 30 min, avoiding stimulants like caffeine, especially before their bedtime, avoiding heavy foods like fatty, fried, spicy foods, and also citrus fruits, and finally assuring sufficient exposure to natural sunlight [2, 3]. Nevertheless, both inadequate and excessive sleep have been regularly reported to be correlated with several health-related conditions like hypertension, obesity, diabetes mellitus [4–6], cardiovascular disorders, stroke, and mortality [5, 7–9].

Metabolic syndrome (MetS) is defined as the group of cardiometabolic risk factors that consists of at least three of the following conditions: high triglyceride levels, raised waist circumference, hypertension, low high-density cholesterol levels, and elevated fasting glucose. Moreover, it is associated with an enhanced risk of cardiovascular disorders and diabetes mellitus [10]. The estimated prevalence of MetS is believed to be approximately one-quarter of the world's population, and therefore it is considered a significant public health challenge [11]. The recent meta-analysis of the Iranian population revealed that at least one-fourth of the population had MetS in Iran [12]. Additionally, the result of Bandare-Kong Non-Communicable Diseases (BKNCD) Cohort Study showed that nearly 34.6% of the population had MetS [13]. According to the survey data from 2003 to 2012 gathered by the United States National Health and Nutrition Examination, the overall prevalence of metabolic syndrome was 33%. They anticipated that around 35% of adults and 50% of individuals aged 60 years or higher had MetS [14]. Based on the data from 9 studies done among European populations, it has been proposed that 38% of women and 41% of the men had MetS [15].

Sleep has an essential role in the homeostasis maintenance of the internal environment, balancing physiological, hormonal, and psychological processes [16–18]. Hormonal changes, if not regulated, may lead to a variety of adverse health conditions, such as diabetes, hypertension, cancer, depression, and even mortality [19–23].

While numerous epidemiological studies have shown a U-shaped relationship between sleep duration and lower health outcomes, however, how many hours precisely are regarded harmful and possible gender discrepancies in the association remain ambiguous [24, 25]. Earlier epidemiologic studies have reported that an increase in sleep duration enhanced the risk of MetS [26–28]; however, those results were inconsistent and limited. This study evaluated the association between sleep duration and MetS based on a cross-sectional survey as the first phase of PERSIAN Cohort Study conducted in Bandare-Kong, a city located in the south of Iran.

## Methods

### Study population

BKNCD provided the data for this descriptive-analytical cross-sectional study. BKNCD (N = 4063) is a large-scale prospective study that took place between November 17, 2016, and November 22, 2018, as part of the Prospective Epidemiological Research Studies in IrAN (PERSIAN) project, which was previously explained in detail [29].

Among subjects, pregnant women and participants using medications causing sleep disorders as well as sedating drugs were excluded. Thus, a total of 3695 subjects were included as the final analytic sample for our study.

### Study design

A face-to-face interview by highly qualified interviewers was conducted using a thorough questionnaire that included demographics, lifestyle variables, eating habits, physical activity, and medical history.

Subjects were weighed on a digital scale (with a measurement accuracy of 0.5 kg) while wearing just the bare minimum of clothes and without shoes. The subject’s height was measured while standing without shoes and with their shoulders positioned naturally. The waist circumference (WC) of each subject was measured twice, and the average was reported. At the end of numerous consecutive natural breaths, the midway between the top of the iliac crest and the inferior border of the last detectable rib in the midaxillary line at a level parallel to the floor was measured. The maximum circumference of the buttocks was measured at a parallel level to the floor, and the hip circumference (HC) was calculated. To the closest 0.5 cm, all measurements were taken with the same stretch-resistant tape. Subjects stood upright during the measurements, with arms relaxed at the side, feet evenly spread apart, and body weight evenly distributed. The waist-to-hip ratio (WHR) was calculated as WC divided by HC to the nearest 0.01.

MetS was defined according to Iranian obesity association guideline when participants met three or more of the following criteria [30]:

1. Abdominal obesity: WC ≥ 95 cm for men and women
2. Triglycerides (TG) ≥ 150 mg/dL or treatment for hypertriglyceridemia.
3. High-density lipoprotein cholesterol (HDL-C) < 40 mg/dL in men and < 50 mg/dL in women
4. Systolic blood pressure (SBP) ≥ 135, diastolic blood pressure (DBP) ≥ 85 mmHg or treatment for hypertension
5. Fasting plasma glucose (FPG) ≥ 100 mg/dL or treatment for elevated fasting glucose

Blood pressure (BP) was measured in the sitting posture, with feet on the floor and arm at heart level, using a standard mercury sphygmomanometer with a cuff size appropriate for the upper-arm circumference, after 5 min of rest. The average of two measurement data taken at least 5 min apart was used in the study.

After an overnight fast of 8-h, venous blood samples were taken, and FPG was measured using the glucose oxidase method. After a 12-h overnight fast, venous blood samples were obtained the next day TG, and HDL-C levels were measured using an enzymatic method.

Our study’s final model was set according to the following covariates which, all were categorized in the same way as the BKNCD: sex, age, education, and marital status. Education had three categories: less than 6 years, 6–12 years, and more than 12 years. Marital status also had three categories: married, single, and widowed/divorced.

Further lifestyle covariates were also considered, such as smoking, alcohol consumption status, and physical activity. The International Physical Activity Questionnaire was used for the assessment of physical activity [31]. The weekly average of 24-h physical activities including leisure time activities, work, and exercise was categorized into 3 groups, namely low physical activity (24–36.5 metabolic equivalent of tasks [METs]), moderate (36.6–44.9 METs), and high (≥ 45 METs) [32]. The sleep status information was collected by surveying the following questions: “On average, how many hours do you sleep per day?” Moreover, sedentary behaviors were derived from: “On average, how many hours do you usually lie, sit, or lean per day?” Sleep duration, the time between going to sleep and waking up, was classified as short sleep: sleep < 7 h, normal sleep: sleep between 7 and 9 h, and long sleep: sleep > 9 h.

### Data analysis

The data were examined, and if the sleep duration value was more than *Q*3 + 3IQR or less than *Q*1 − 3IQR, as an outlier was removed (*Q*1: first quartile, *Q*3: third quartile, IQR: *Q*3 − *Q*1). To have a better smoothing function in generalized additive models (GAMs), people who sleep between 12 and 13 h fall into the 12-h sleep group.

Furthermore, outliers were deleted for the five standard components of MetS (waist circumference, SBP, HDL-cholesterol, TG, and fasting plasma glucose), and MetS severity score were calculated using a confirmatory factor analysis approach for the five components of MetS and were treated as a continuous outcome. Moreover, the existence of MetS was considered as a categorical outcome.

Due to the existence possibility of a U-shape association between MetS and sleep duration, as seen in previous studies, the GAMs were used [33].

In the present study, six models of GAM were employed. The MetS (yes/no) was considered a binary response variable for the first three models, whereas the fourth and fifth models MetS severity score as a continuous response variable was used.

Response predictor

- Model 1: Metabolic syndrome (yes/no) smooth (sleep).
- Model 2: Metabolic syndrome (yes/no) smooth (sleep) for different sexes.
- Model 3: Metabolic syndrome (yes/no) age, sex, sitting, smooth (sleep).
- Model 4: Metabolic syndrome (yes/no) age, sitting, smooth (sleep * sex).
- Model 5: Metabolic syndrome severity score sex, smooth (sleep).
- Model 6: Metabolic syndrome severity score age, sex, sitting, smooth (sleep).
- Model 7: Metabolic syndrome severity score age, sitting, smooth (sleep * sex).

In model 1, MetS (yes/no) was used as a binary response and a smoothing function of sleep duration as a univariable predictor. Model 2 is the same as Model 1 which has been run in different sexes separately. Model 3 was a multivariable version of model 1; it used binary MetS (yes/no) as a response, but the predictor variables are age, sex, sitting time, smooth (sleep). Model 4, likewise model 3, but the interaction between sex and sleep duration was added. In model 5, the MetS severity score was used as the continuous response, sex, and smooth (sleep) as predictors. Model 6 is the same as Model 3; just MetS severity score was applied as the continuous response; it used age, sex, sitting time, smooth (sleep) as predictors. The last model was similar to model 6; however, the smoothing function of the interaction between sex and sleep duration was added to the model.

Since there was a high correlation between BMI and WC using Pearson correlation analysis, then BMI was not considered as a variable in the models.

A generalized additive model (GAM) is a generalized linear model in which relationships between the predictors and the dependent variable follow smooth patterns that can be linear or non-linear. Age, sex, and sitting time were used to adjust GAMs.

The values of effective degree of freedom (EDF) demonstrate the degree of curvature of the smooth. A value of 1 indicates a linear pattern of relationship. A value of EDF > 1 shows a more complex relationship between MetS and sleep duration. A P < 0.05 was considered as a statistically significant level, and data was analyzed by R software.

## Results

A total of 3695 participants, aged 35–70, were included in the analyses. The mean age was 48.05 years (SD 9.36), and 2067 (55.9%) were female. Overall, 58.6% of the participants' education level was below the middle school, and 89.9% were married. The mean sleep duration was 7.20 (SD 1.46) hours. Furthermore, the estimated Prevalence of MetS was 35.9%, and women appeared to be more likely to have MetS than men (P < 0.001).

More detailed characteristics of the participants, according to metabolic syndrome existence, are presented in Table 1.

**Table 1:** Characteristics of participants based on metabolic syndrome

Participants’ characteristics	Metabolic syndrome		
	No, n = 2367	Yes, n = 1328	p
Age, years, mean (SD)	46.2 (8.9)	51.2 (9.2)	< 0.001
Sex, n (%)			
Male	1224 (33.1)	404 (10.9)	< 0.001
Female	1143 (30.9)	924 (25.1)	
Marital Status, n (%)			
Single	63 (1.7)	25 (0.7)	< 0.001
Married	2156 (58.3)	1167 (31.6)	
Widowed/divorced	148 (4.0)	136 (3.7)	
Education, n (%) (years)			
< 6	1244 (33.7)	923 (24.9)	< 0.001
6–12	882 (23.9)	328 (8.9)	
> 12	241 (6.5)	77 (2.1)	
Sitting, hours/day, mean (SD)	9.5 (2.5)	10.5 (2.6)	< 0.001
Sleep duration, hours, mean (SD)	7.2 (1.4)	7.1 (1.5)	0.2
Diastolic blood pressure, mmHg, mean (SD)	74.6 (9.5)	81.1 (10.6)	< 0.001
Systolic blood pressure, mmHg, mean (SD)	113.9 (14.5)	127.0 (18.9)	< 0.001
Fasting blood glucose, mg/dL, mean (SD)	96.5 (27.7)	128.2 (55.7)	< 0.001
High density lipoprotein, mg/dL, mean (SD)	49.4 (10.4)	45.1 (10.8)	< 0.001
Triglyceride, mg/dL, mean (SD)	113.8 (60.7)	177.0 (112.2)	< 0.001
Waist circumference, cm, mean (SD)	90.1 (11.2)	99.9 (10.2)	< 0.001

SD: standard deviation

**Figure1:**
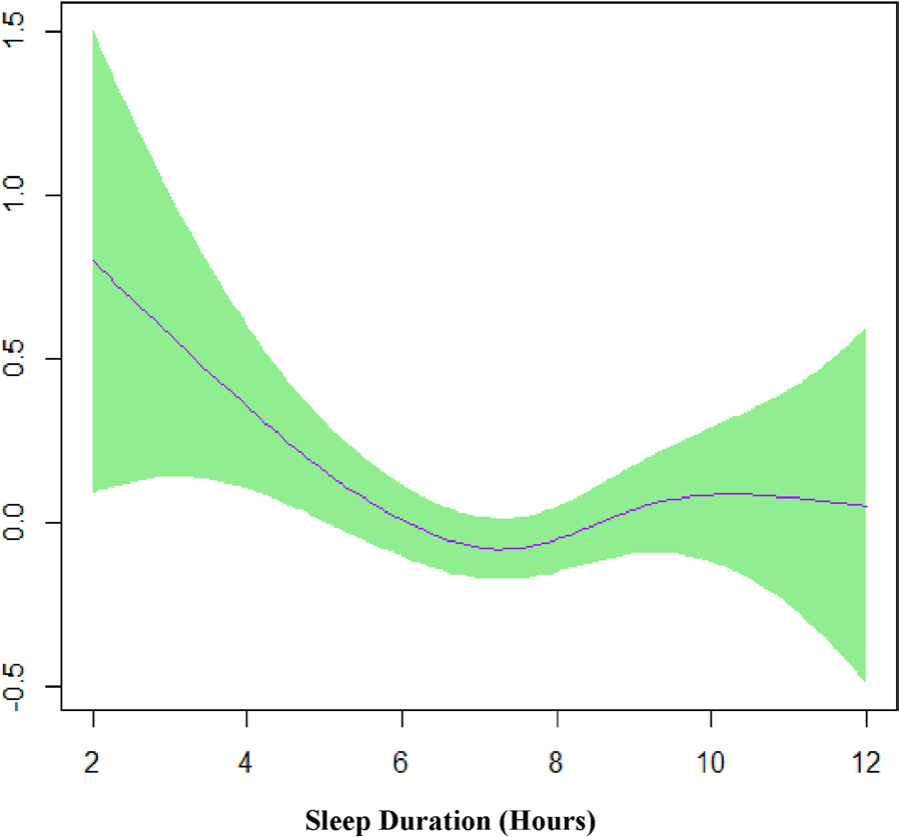
Model 1 shows the univariable smooth function of sleep duration with 95% confidence band (EDF = 3.09, p = 0.02)

Figure 1 illustrates the plots of the estimated smooth function for sleep duration with a 95% confidence band for model 1 based on GAMs analysis. Figure 1 confirms the non-linear association of sleep duration and the risk of MetS in the univariate GAMs. The lowest risk is observed among those who had 7–7.5 h of sleep duration per night.

**Fig. 2:**
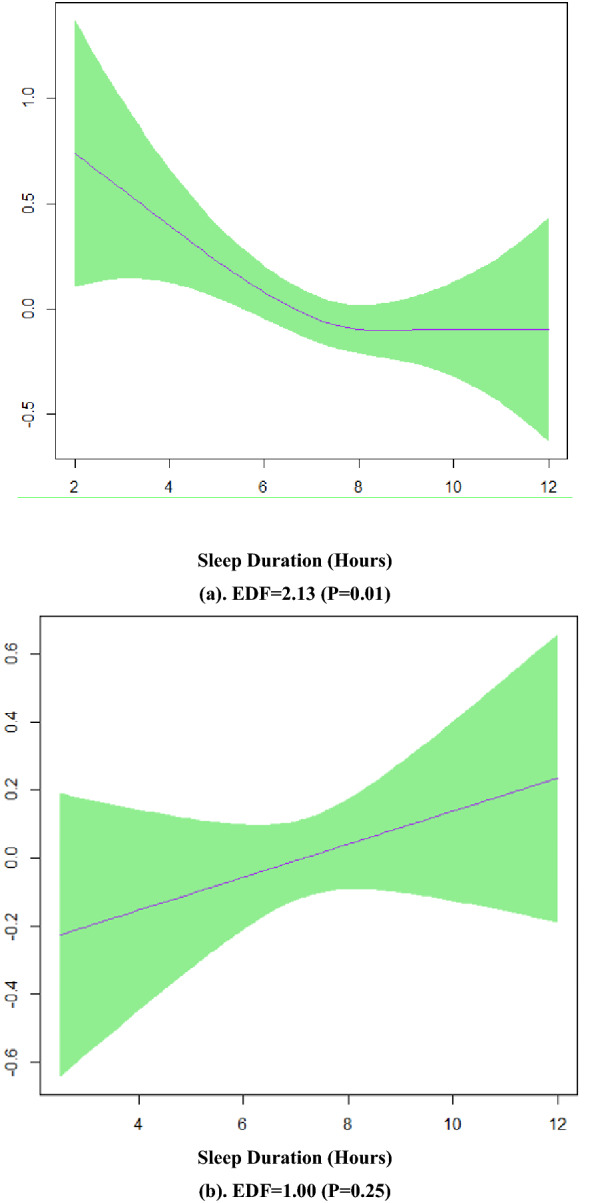
**a** Smooth function of sleep duration with 95% confidence band for females in model 2, **b** model 2 for males

Figure 2 shows the plots of the predicted smooth relationship between MetS (yes/no) and sleep duration separately for males and females in model 2. This figure confirms a non-linear association of sleep duration and MetS in females and a linear pattern in males.

**Figure3:**
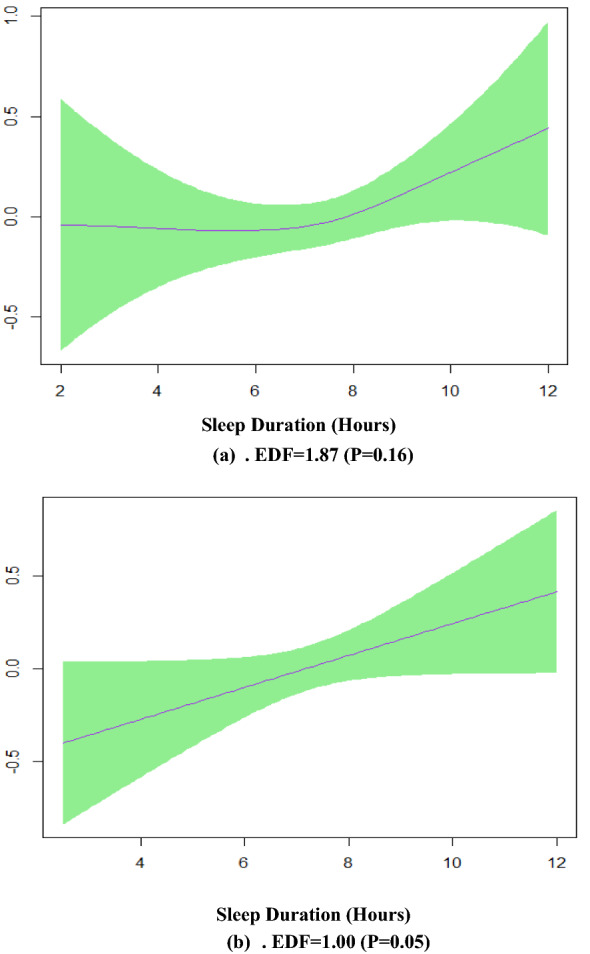
Effect modification of sex and sleep duration in the multivariable generalized additive model 4 when the response variable was MetS. **a** Smooth function of sleep duration with 95% confidence band for females, **b** for males

Table 2 indicates the relationship between MetS and independent variables in model 3 by GAMs output. Based on Table 2, an EDF of 1.49 indicates a semi-linear fit between sleep duration and MetS. Model 4 represented two different smoothing shapes; an almost U-shaped in women and a linear one in men (Fig. 3).

**Table 2:** Association of metabolic syndrome and independent variables measured by multivariable generalized additive model 3

R^2^ = 0.121	Outcome: metabolic syndrome	
n = 3695	β	P
Age	0.06	< 0.001
Sex		
Male	Reference group	
Female	0.91	< 0.001
Sitting	0.08	< 0.001
Sleep	Smooth curve, EDF = 1.49	0.114

**Fig. 4:**
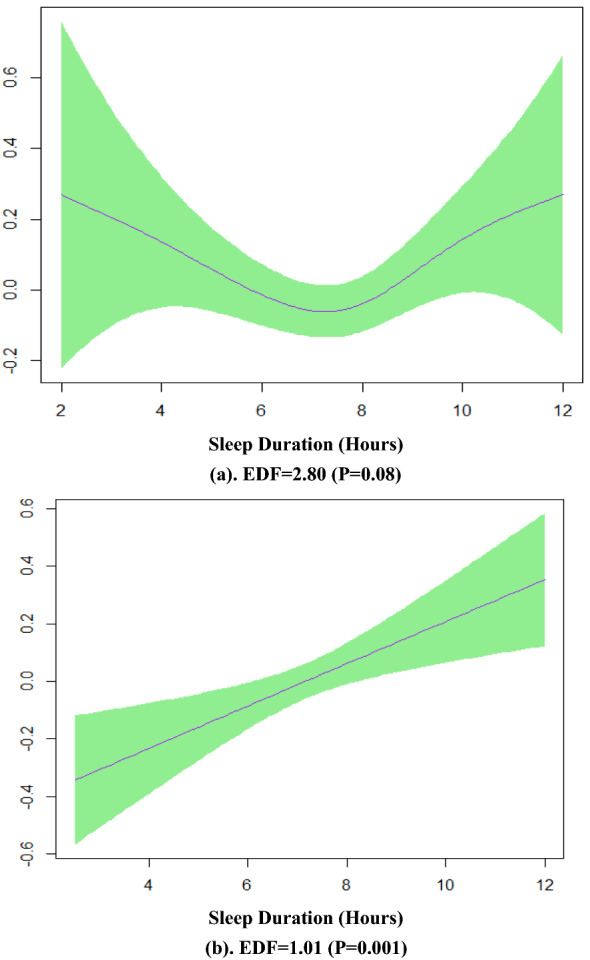
**a** Smooth function of sleep duration with 95% confidence band for females in model 5, **b** model 5 for males

Figure 4 presents the plots of the predicted smooth relationship between MetS severity score and sleep duration separately for males and females in model 5. This figure confirms a U-shape association of sleep duration and MetS severity score in females and a linear pattern in males.

Table 3 shows the output of model 6, a significant EDF of 1.85 reveals a non-linear pattern between sleep duration and MetS severity score. A significant EDF of 1.85 indicates an almost non-linear fit between sleep duration and MetS severity score.

**Table 3:** Association of metabolic syndrome and independent variables measured by model 6

R^2^ = 0.06	Outcome: metabolic syndrome severity score	
n = 3695	β	P
Age	0.02	< 0.001
Sex		
Male	Reference group	
Female	0.37	< 0.001
Sitting	0.06	< 0.001
Sleep	Smooth curve, EDF = 1.85	< 0.001

**Fig. 5:**
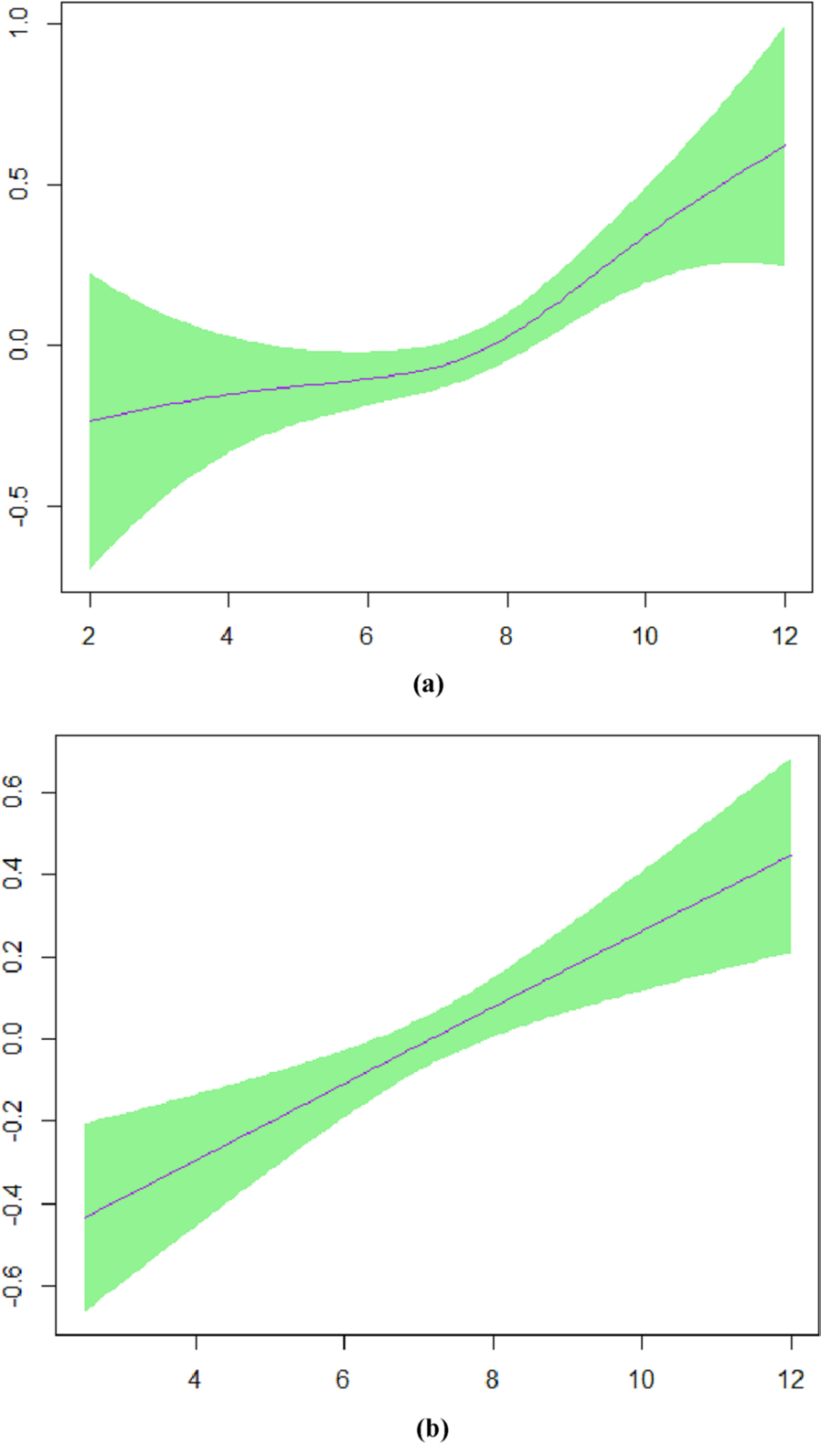
Effect modification of sex and sleep duration in the multivariate generalized additive model 7 when the response variable was metabolic syndrome severity score. **a** Smooth function of sleep duration with 95% confidence band for females, **b** for males

Furthermore, the multivariate GAM model 7 shows two different smoothing patterns for metabolic syndrome severity score; a non-linear fit in women (EDF = 2.70, p < 0.001) and a linear pattern in men (EDF = 1.02, p < 0.001) (Fig. 5).

## Discussion

To the best of our knowledge, this is the first survey of the BKNCD Cohort Study database that examined the association between sleep duration and risk of MetS among males and females separately in the south of Iran. Our research presented the most up-to-date estimates of MetS prevalence among people aged 35 to 70. MetS was projected to affect 35.9% of the population, with women being more affected than men. The current cross-sectional analysis found statistically significant associations between short and long sleep periods and MetS risk, supporting the existence of a U-shape relationship in women and a linear association in men.

According to the univariate model, people who slept 7–7.5 h a night have the lowest chance of MetS. Similarly, those who slept less than or more than 7.5 h had a higher risk than those who slept for 7 h.

The current study showed that long sleep was associated with MetS in both genders. In our research, the gender interaction in the relationship between sleep duration and MetS was statistically significant; however, a similar study finding displayed that long sleep was associated with MetS in women only [34].

Several mechanisms for MetS associated sleep disorders have been proposed, including endocrinologic, immunologic, biologic, hormonal, and metabolic processes. Sleeping for less than 7 h can provoke mutual changes in circulating leptin and ghrelin levels, resulting in high ghrelin and low leptin levels [35], increasing appetite, caloric intake, and lowering energy expenditure [30, 36], facilitating an increase in central obesity, one of the main components of MetS. Sleep deprivation has also been associated with decreased glucose tolerance, associated with an increased risk of hypertension and diabetes [37]. Increased cortisol levels, which can raise fasting glucose levels, are another endocrinologic consequence of sleep restriction [38]. In addition, clinical studies have shown that sleep deprivation increases levels of Interleukin 6 (IL-6) and C-reactive protein (CRP), both of which have been linked to MetS constituents through increased insulin resistance [39].

Circadian regulation of energy metabolism and hormonal secretion are the possible causal mechanism [16, 17, 39]. Sleep disruption has been linked to impaired metabolism and appears to play a role in the pathogenesis of metabolic diseases. Circadian rhythmicity and autonomic balance can be disrupted due to sleep timing and duration changes, resulting in diurnal cardiac output rhythm disturbance and increased blood pressure variability [40, 41].

Among other potential mechanisms of sleep-MetS interaction, it is worth noting that stage 3 of sleep is the most important since this is when the growth hormone (GH) and GH releasing hormone (GHRH) are released. They provoke fat loss, bone growth, and general repair and regeneration. Before midnight, the longest part of stage 3 sleep occurs. Sleep deprivation will inhibit the most potent GH pulse, raising the risk of MetS [33].

Only a few studies have documented gender-stratified sleep associations with MetS is one of the current study’s key strengths. A meta-analysis consisting of 12 cross-sectional and three cohort studies from North America, Europe, and Asia found that sleep duration of fewer than 5 h and greater than 8 h were both linked to MetS but with no gender differences [28].

Furthermore, we were able to support the idea that both short and long sleep are linked to MetS in women in our research, but a recent meta-analysis, while finding a dose–response association between short and long sleep and MetS, was unable to support the idea that long sleep is linked to MetS as well [42]. Our findings indicated a linear association between sleep duration and metabolic syndrome in men. On the other hand, long sleep duration increased the risk of metabolic syndrome and metabolic syndrome severity score while short sleep duration did not affect. A Korean study found that a sleep duration greater than or equal to 9 h was linked to MetS, but no connection was found between short sleep and MetS [43].

Among the studies’ other strengths, we can point to the analysis process and the fact that the MetS severity score was used. Using the MetS severity score improved the association’s strength because it provided a continuous measure of the risk of metabolic status, whereas MetS definition using qualitative criteria is not an accurate measurement compare with MetS score. Furthermore, all five elements of the MetS, whether high, borderline, or low, contribute to the MetS severity score measurement. In contrast, only the high components identified by the threshold specified in the definition are considered to diagnose MetS [44]. This limitation could be resolved by measuring the MetS severity score, including the actual measurements of all five components. Other studies have shown the accuracy of the MetS severity score in estimating the likelihood of health outcomes [45, 46]. Furthermore, the generalized additive models’ application to investigate the relationship between sleep and MetS and MetS severity score improved the risk adjustment compared to other models [47, 48].

While our research shows a connection between sleep duration and MetS, it also has several limitations. First, as the current study is a cross-sectional analysis, it restricts us from supposing the causative link between sleep duration and MetS. Hence, this study should be conducted jointly into a prospective cohort study.

Second, rather than using objective measurements like an actigraph or polysomnography, sleep duration was measured using self-report questionnaires. As a result, knowledge bias may occur based on how much sleep one believes they got [49].

Third, the total amount of sleep time measured may include both nighttime and naptime sleep. Since daytime napping has previously been linked to lower sleep quality, shorter sleep length, and, as a result, cardiovascular risk factors [50], it would be beneficial to differentiate between naptime and nighttime sleep to determine their effect on health separately.

Fourth, data on sleep quality, sleep disruptions, insomnia symptoms as well as sleep apnea were not analyzed because they were not available. Sleep disruptions have been linked to metabolic disorders in previous studies [51], highlighting the significance of considering sleep quality when assessing the impact of sleep on overall health.

Eventually, longitudinal studies in different populations could help to improve the reliability and generalizability of this study’s findings.

## Conclusion

Findings represented a linear pattern between the risk of MetS as well as higher MetS severity score and sleep duration in men and an almost u-shaped pattern in women. Of note of the growing prevalence of metabolic syndrome and its role as a significant risk factor of cardiometabolic disease, the sleep disorder should be considered a health priority to reduce and improve metabolic syndrome. It would be suggested to conduct longitudinal studies to evaluate the relationship between sleep quality components, including sleep duration and MetS in the next phases of the study.

## Data Availability

The datasets used and/or analyzed during the current study are available from the corresponding author on reasonable request.

## Abbreviations

PERSIAN: Prospective Epidemiological Research Studies in IrAN
BKNCD: Bandare-Kong Non-Communicable Diseases
ADA: American Diabetes Association
MetS: Metabolic syndrome
ATP III: Adult Treatment Panel III
BMI: Body mass index
CVD: Cardiovascular disease
BP: Blood pressure
DBP: Diastolic blood pressure
SBP: Systolic blood pressure
FPG: Fasting plasma glucose
HC: Hip circumference
TC: Total cholesterol
WC: Waist circumference
WHO: World Health Organization
WHR: Waist-to-hip ratio
TG: Triglyceride
HDL: High-density lipoprotein
LDL: Low-density lipoprotein
METs: Metabolic equivalent of task
GAMs: Generalized additive models
OR: Odds ratio
CI: Confidence interval
GH: Growth hormone
GHRH: Growth hormone-releasing hormone
CRP: Creactive protein
